# Changes in Physical Activity and Sedentary Behavior Amid Confinement: The BKSQ-COVID-19 Project

**DOI:** 10.2147/RMHP.S268320

**Published:** 2020-09-25

**Authors:** Mahmoud A Alomari, Omar F Khabour, Karem H Alzoubi

**Affiliations:** 1 Department of Physical Education, Qatar University, Doha, Qatar; 2 Department of Medical Laboratory Sciences, Jordan University of Science and Technology, Irbid, Jordan; 3 Department of Clinical Pharmacy, Jordan University of Science and Technology, Irbid, Jordan

**Keywords:** physical activity, sedentary behavior, confinement, COVID-19, pandemic

## Abstract

**Background:**

Coronavirus disease 19 (COVID-19) has compelled implementing confinement measure across the globe. These measures can potentially lead to many changes in lifestyle. However, no studies examined the effect of COVID-19-induced confinement on physical activity (PA) and sedentary behavior (SB).

**Methods:**

During April and May of 2020, the current study surveyed changes in PA and SB induced by COVID-19 confinement.

**Results:**

The participants of the study were 1844. Among the participants who were regularly involved in PA, the majority (41.8–42.2%) of the participants reported a “decrease” (*p*<0.05) in walking, jogging, and sports while the majority (46.3–53.1%) reported a “no change” (*p*<0.05) in swimming, cycling, and weight lifting. With regard to the SB, most of the participants reported an “increase” in watching TV (72.3%), using electronics (82.7%), and logging to social media (81.9%). Additionally, gender, job type, obesity, and being worried to contract the disease were associated (*p*<0.05) with changes in PA. On the other hand, age, gender, obesity, job type and income were related (*p*<0.05) to changes in SB.

**Conclusion:**

Results of the current study might enhance knowledge about the impact of COVID-19 on lifestyle, particularly PA and SB. Subsequently, it can also be used to establish strategies to enhance engagement in activities during the current and future pandemics.

## Introduction

Coronavirus disease 19 (COVID-19) started in China, Wuhan in November of 2019 and was declared as a fatal pandemic by the WHO in March, 2020.^1^,^2^ According to WHO data, millions of COVID-19 cases leading to hundreds of thousands fatalities have been documented worldwide, as of June of 2020.

The COVID-19 is a highly pathogenic single-stranded RNA virus that belongs to the genus Beta coronavirus.^3^–^6^ Human-to-human transmission of the COVID-19 is usually via direct contact and is higher among individuals in one household.^7^,^8^ Commonly reported symptoms included cough, fever, breathing difficulty, sore throat, headache, vomiting and diarrhea.^9^ Though is experienced less frequently, the most potent effect of the virus is attacking the respiratory track cells via its envelope proteins.^10^,^11^ Subsequently, some patients develop acute respiratory failure and sepsis, which are considered the major complications of the disease.^12^,^13^

As a response to the spread and devastating effect of the virus, several actions have been taken. These actions include suspension of schools, banning public gatherings, closure of the borders, airports, businesses, and worship places, and restriction of local travel. Such actions have been implemented across the globe with slight differences between countries. Subsequently, people spend most of their time at home, which is expected to impact most aspects of their routine lifestyle such as physical activity (PA) and sedentary behavior (SB). A recent study among adults in the UK showed limited physical activity experienced during the application of COVID-19 related social distancing measures.^14^ Another study from Brazil showed reduced moderate to vigorous physical activity and increased SB during the application of COVID-19 related social distancing measures.^15^

Such changes in lifestyle during the COVID-19 crisis might have long-term effects on health, subsequently the body response to infections. In a review, exercise has been shown to ameliorate the response of the body to respiratory tract viral infections. The review concluded that moderate-intensity PA enhances immune function and reduces the risk and severity of respiratory viral infections. Exercise seems to elevate stress hormones, subsequently enhance the Th2 immune response pathway.^16^ Another review reported that engaging in moderate PA may enhance the immune function, especially in less ﬁt subjects/sedentary population. On the other hand, intense exercise may cause immunosuppression and increases the risk of respiratory viral infection.^17^ Thus, changes in lifestyles during COVID-19 crisis, especially PA and SB might have health consequences on the response of the body to respiratory tract infections. Therefore, in the current investigation, changes in PA and SB during COVID-19 crisis were investigated. The results of this study might enhance current knowledge about the impact of COVID-19 on lifestyle, particularly PA and SB. Subsequently, highlights the needs for interventions to prepare for the negative effects of the current and possible future calamities.

## Methods

### Design and Participants

The current data is derived from a larger project, the “Behavior, Knowledge, Stress and Quality of Life during COVID19-induced Confinement (BKSQ-COVID19) project”. The study is a cross-sectional survey distributed during April and May of 2020 to examine changes in PA and SB. Jordanian adults (age >18 years) of both genders from most parts of Jordan were invited to participate in the study. The questionnaire was distributed anonymously and electronically via social media platforms. Participants were informed about the objectives of the study and consented electronically prior to filling the questionnaire. Institutional Review Board (IRB) of Jordan University of Science and Technology approved the study procedures.

### Questionnaire

Given the novelty of the disease, no previous questionnaires were appropriate enough to fit the objectives of the study. Thus, the research team developed the questionnaire based on similar studies.^18^ The questionnaire covers domains including demographics, perceptions about COVID-19 disease, and changes in PA and SB during the pandemics. Demographic parameters included age, gender, weight, height, job type (ie educational, medical, versus managerial), education, and income. Additionally, COVID-19 perception and confinement information, such as the likelihood of getting infected, knowing somebody infected with COVID-19, and the implemented governmental confinement procedures, were surveyed.

The examined PA measures were walking, jogging, swimming, sporting, weight lifting, while watching TV, using electronics, and logging to social media were considered SB. The participants were asked “What changes have you experienced in the following lifestyle behaviors due to the spread of coronavirus?”. Four choices were available, “increase”, “decrease”, “no change”, and “never practiced this behavior”.

### Statistics

The SPSS (version 21) was used to statistically analyze the data. For statistical significance, α was set a priori at 0.05. The data are presented in mean±SD, frequency and percentages. The Chi^2^ goodness of fit test was used to determine the differences in the distribution of the participant responses, “increase”, “decrease”, versus “no-change” in PAs and SBs. Cross-tabulation was used to determine the relationship of potential factors with the participant responses to the questions about the changes in PAs and SBs, “increase”, “decrease”, or “no-change”. The factors found, using cross-tabulation, related to the responses to each question were incorporated into a multinomial model to determine the ability of each factor to predict the responses. The potential factors were age, gender, obesity (BMI classifications), income, education, job type, worried to be infected COVID-19, and knowing a person infected with COVID-19.

## Results

### Participants

The participants’ characteristics are presented in Table 1. A total of 1844 individuals responded to the questionnaire. Age, weight, and height ranges were 18–72 years, 38–144 kg, and 120–198 cm. Most of the participants were women, with a bachelor's degree, receiving middle income, who are unemployed while more than 50% were either overweight, obese or severely obese. As in Table 2, very few participants were worried about getting infected or knew a person who was infected. Additionally, the majority of the participants reported a range of confinement practices and advisories, including self-quarantine, social distancing, lockdown, school closure, and event banning.

**Table 1:** The Participant Demographic (n=1844)

Gender (%; male)		30.5
Age (yrs, mean±SD)		33.7 ± 11.3
Weight (kg, mean± SD)		72.6± 16.3
Height (cm, mean±SD)		166.3± 9.0
Obesity (BMI; %)		
	Under weight	2.1
	Normal weight	43.3
	Overweight	35.4
	Obese	14.8
	Overly obese	4.4
Level of education (%)		
	High school and less	19.4
	Associate degree	14.1
	Bachelor degree	51.3
	Graduate degree	15.3
Income (%)		
	Low	34.5
	Middle	65.5
	High	
Job type (%)		
	Unemployed/retired	35.6
	Military/police	4.8
	Education	23.9
	Agriculture	1.8
	Health	14.0
	Manufacturing	2.8
	Engineering	5.8
	Management	8.2
	Crafting	3.2

**Table 2:** Perception and Confinement Information Related to COVID-19 (n=1844)

Likelihood of getting infected		(%)
	Low	59.5
	Moderate	34.5
	High	6.0
**Know somebody who is infected**		
	Yes	6.3
	No	93.7
**Self-quarantine**		
	Yes	93.5
	No	6.5
**Physical distancing**		
	Yes	96.8
	No	3.2
**Banning group events (ie weddings)**		
	Yes	98.2
	No	1.8
**School closure**		
	Yes	99.0
	No	1.0
**Lockdown**		
	Yes	97.0
	No	3.0

### Prevalence of Physical Activities and Sedentary Behaviors

As in Table 3, the majority of the participants reported no involvement in PA including cycling (71.4%), swimming (65.2%), sports (56.4%), and weight lifting (68.2%). Additionally, most of the subjects were involved in all the surveyed SB including watching TV (94.6%), using electronics (97.6%), and logging to social media (99.2%).

**Table 3:** Prevalence of Physical Activities and Sedentary Behavior

	Participation	No Participation
**Physical activities**		
Walking	92.8	7.2
Jogging	79.9	20.1
Swimming	34.8	65.2
Cycling	28.6	71.4
Sports	43.6	56.4
Weight lifting	31.8	68.2
**Sedentary behavior**		
Watching TV	94.6	5.4
Using electronics	97.6	2.4
Logging to social media	99.2	0.8

**Note:** Values presented are the percent (%) of participants.

### Changes in Physical and Sedentary Activities

The chi-square goodness-of-fit test demonstrates differences (*p*<0.05) between the participants’ responses to the questions about participating in PAs and SBs, “increase”, “decrease”, versus “no-change”. Table 4 shows that among the participants who were regularly involved in PA, the majority of the participants reported a decrease in walking, jogging, and sports while the majority reported a no change in swimming, cycling, and weight lifting. With regard to the SB, most of the participants reported an increase in watching TV (72.3%), using electronics (82.7%), and logging to social media (81.9%).

**Table 4:** Changes in Physical Activities and Sedentary Behavior

Activities	Decreased	No Change	Increased	χ^2^ *p*-value
**Physical**				
Walking	42.2	23.9	33.8	85.5; 0.0001
Jogging	41.8	37.2	21.0	101.2; 0.0001
Swimming	44.5	49.0	6.5	90.5; 0.0001
Cycling	26.6	53.1	20.3	195.3; 0.0001
Sports	41.6	39.9	18.9	70.9; 0.0001
Weight lifting	35.9	46.3	17.9	68.1; 0.0001
**Sedentary**				
Watching TV	5.6	22.1	72.3	1210.8; 0.0001
Using electronics	3.2	14.0	82.7	1932.5; 0.0001
Logging to social media	3.0	15.1	81.9	1916.9; 0.0001

**Note:** Values presented are the percent (%) of participants.

### Factors Contributing to the Changes in Physical Activities

Cross-tabulation tests were used to examine the relationship of PA measures with potential contributing factors. Values are expressed as percentage. Walking was related to gender (χ^2^=7.3; *p*=0.026) and job type (χ^2^=38.7; *p*=0.001) while jogging was related to age (χ^2^=19.0; *p*=0.001) and job type (χ^2^=30.7; *p*=0.015). The cross-tabulation also revealed that cycling was related to age (χ^2^=19.9; *p*=0.001) while swimming was related to age (χ^2^=11.8; *p*=0.02) and obesity (χ^2^=15.9; *p*=0.04). Additionally, the analysis showed a relationship of sports with age (χ^2^=14.0; *p*=0.007) and gender (χ^2^=25.9; *p*=0.0001) as well as an association of weight lifting with age (χ^2^=20.8; *p*=0.0001) and obesity (χ^2^=17.9; *p*=0.02).

### Factors Contributing to the Changes in Sedentary Behaviors

The cross-tabulation revealed a relationship between TV watching time and age (χ^2^=13.8; *p*=0.008), gender (χ^2^=9.1; *p*=0.01), obesity (χ^2^=19.9; *p*=0.01), and income (χ^2^=15.7; *p*=0.003). The cross-tabulation showed that electronic use was related to education (χ^2^=15.6; *p*=0.016), income (χ^2^=16.5; *p*=0.014), and job type (χ^2^=46.9; *p*=0.0001).

### Predictors of the Changes in Physical Activities

The factors that were found related to each perspective PA parameter using cross-tabulation, were added to the regression model. Additionally, the “no change” choice was used as the reference for all PA variables in the regression model. The regression showed that the combination of gender and job type can predict participating in walking (χ^2^= 40.8; *p*<0.002; NagR^2^=0.03). However, further analysis showed that only job type contributes meaningfully to the model (χ^2^= 36.0; *p*=0.003). The participants holding a job in the military (β=−1.0; OR=0.35; *p*=0.035), agriculture (β=−1.4; OR=0.25; *p*=0.024), health (β=−1.1; OR=0.31; *p*=0.006), and engineering (β=−1.1; OR=0.34; *p*=0.02), versus a job in crafting, were less likely to select an “increase” in walking versus “no change”.

The regression showed that the combination of age and job type can predict participating in jogging (χ^2^=29.0; *p*<0.0001; NagR^2^=0.04). Additional analysis revealed that age (χ^2^= 16.7; *p*=0.002) and job type (χ^2^= 30.0; *p*=0.02) can each contribute uniquely to the model. The younger individuals, versus the elderly, were more likely to select a “decrease” (β=0.6; OR=1.8; *p*=0.007) and an “increase” (β=0.5; OR=2.4; *p*=0.02) in jogging versus a “no-change”. The regression showed that age can predict participating in cycling (χ^2^=19.5; *p*<0.001; NagR^2^=0.45). Further analysis, however, showed that age can contribute uniquely to the model with middle age, comparing to elderly, more likely to select a “decrease” (β=0.6; OR=2.0; *p*=0.05) in cycling versus a “no-change”.

The regression showed that the combination of age and obesity can predict participating in swimming (χ^2^=22.6; *p*<0.03; NagR^2^=0.05). Further analysis, however, showed that neither age (χ^2^=6.5; *p*>0.16) nor obesity (χ^2^=10.0; *p*>0.26) can uniquely contribute to the model. The regression showed that the combination of age and gender can predict participating in sports (χ^2^=42.4; *p*<0.0001; NagR^2^=0.06). Further analysis showed that age (χ^2^=13.9; *p*=0.007) and gender (χ^2^=28.9; *p*=0.0001) can each contribute uniquely to the model. Being younger (β=0.7; OR=2.0; *p*=0.007), versus elderly, while men (β=0.4; OR=1.5; *p*=0.017) versus women, is more likely to select a “decrease” while being a man (β=−0.7; OR=0.5; *p*=0.001), versus being a woman, is less likely to select an “increase” in sport participation.

The regression showed that the combination of age and obesity can predict (χ^2^= 26.6; *p*<0.0001; NagR^2^=0.07) participating in weight lifting. Further analysis, however, showed that only age (χ^2^= 15.5; *p*=0.004) can contribute uniquely to the model. Being younger (β=0.7; OR=2.0; *p*=0.05), versus elderly is more likely, while being obese (β=−1.1; OR=0.3; *p*=0.04), versus overly obese, is less likely to select a “decrease” in weightlifting.

### Predictors of the Changes in Sedentary Behavior

The factors were found related to each perspective SB parameter using cross-tabulation, were added to the regression model. Additionally, the “no change” choice was used as the reference for all SB variables in the regression model. The regression showed that the combination of age, gender, obesity, and income can predict participating in TV watching time (χ^2^= 55.8; *p*<0.0001; NagR^2^=0.044). Additional analysis showed that age (χ^2^= 13.0; *p*=0.01), obesity (χ^2^= 21.0; *p*=0.007), and income (χ^2^= 15.7; *p*=0.003) can each contribute uniquely to the model. Being younger (β=0.6; OR=0.2; *p*=0.02), versus older, is more likely, while being normal (β=−1.3; OR=0.3; *p*=0.007) and over (β=−1.5; OR=0.2; *p*=0.003) weight, versus overly obese, was less likely to select a “decrease” in TV watching time. Additionally, being a man (β=0.3; OR=1.3; *p*=0.04), versus a woman, was more likely to select an “increase” in TV watching time. The regression showed that the combination of education, income, and job type can predict participating in electronic use (χ^2^= 62.9; *p*<0.0001; NagR^2^=0.06). Additional analysis showed that only job type (χ^2^= 41.8; *p*=0.0001) can contribute uniquely to the model.

## Discussion

The study examined the changes in PAs and SBs during the COVID-19-induced confinement. The results showed that the majority (41.8–42.2%) of the participants reported a decrease in walking, jogging, and sports while the majority (46.3–53.1%) reported a no change in swimming, cycling, and weight lifting. Conversely, the majority (72.1–82.7%) of the participants reported an “increase” in all of SBs. Subsequent analysis indicates that walking was related to gender and job type and jogging was related to age and job type while cycling was related to age and swimming was related to age and obesity. Sport was related to age and gender while weightlifting was related to age, obesity, and being worried to conduct the disease. Watching TV was related to age, gender, obesity and income while using electronics was related to the job type. Given the lack of studies, the present study provides unique information to understand the changes in PAs and SBs during COVID-19. The results can be used to establish strategies to enhance engagement in activities during the current and future pandemics.

Confinement is often experienced under few settings, prison, Antarctic camps, and space trips. The data from these settings have suggested a range of psychological and physiological symptoms. Among these symptoms are cognitive, neural, and hormonal alterations, lethargy, skewed circadian rhythm, sleeping difficulties, and diminished immune regulations.^19^–^21^ In fact, some studies have found a relationship between confinement and death after release from prison.^22^ Mental health was also shown to be negatively impacted by COVID-19-related confinement leading to increased stress anxiety and depression symptoms.^23^–^26^ Studies have shown that lockdown is a great stress for citizens; thus, the characteristics of exercise selection may be affected by such a stress. A previous review has concluded a differential impact for stress on exercise adoption, maintenance, and relapse, where habitually active individuals exercise more in the face of stress, and those in beginning stages exercise less.^27^ In a study from Italy, the lockdown was also shown to adversely impact dietary habits leading to increased consumption of “comfort food” such as chocolate, ice-cream, and desserts, and salty snacks.^28^

Since the COVID-19 breakout, several reports have cautioned of the possible decrease in PA,^29^–^33^ increase in SB,^34^ and subsequent adverse health effects during COVID-19-induced confinement.^34^ As far as the author's knowledge, one study reported 36.9%, 34.7%, 42.7%, and 38% decrease in vigorous, moderate, walking, and total PA METs, respectively, while SB increased by 5–8 hours/daily.^35^ A recent study from Brazil showed reduced moderate to vigorous physical activity and increased SB during the application of COVID-19 related social distancing measures.^15^ Similarly, a large portion of the current sample reported a decrease in PA and an increase in SB. These results are alarming and might be associated with adverse health effects. These studies have suggested the possibilities of increased risk of cardiovascular,^33^,^34^ metabolic,^34^,^36^ neural,^34^ and muscular^34^ disease symptoms. Additionally, results from inactivity models including bed rest, limb suspension, and step-reduction have demonstrated rapid muscle wasting, fiber denervation, neuromuscular junction damage, suppressed protein synthesis, and augmented protein degradation.^34^,^37^ Reduced PA and increased SB may also impact the metabolic system, including glucose homeostasis and diminished insulin sensitivity. Impaired cardiovascular endurance, subsequent to diminished cardiac and vascular functions and muscular oxidative capacity, has also been reported. Physical inactivity has also been implicated in the positive energy balance and subsequent fat deposition and weight gains.^34^ Fortunately, these negative adverse effects can be ameliorated by regular participation in PA.^38^

The benefits of regular PA are undeniable and unlimited. It decreases the risk, hospitalization, morbidity, and mortality of many noncommunicable diseases. These include diseases of the cardiovascular, metabolic, immune, neural, and hormonal systems. Additionally, it is an essential component of strategies to enhance weight loss and maintenance, mental health, quality of life, and well-being.^38^ More recently, some research is arguing the environmental benefits of PA including generating extra returns of investments, lowering fossil fuel dependence, and less congested and safer roads, and cleaner air, which are shared goals of the ambitious global 2030 Sustainable Development Agenda.^39^

The measured parameter of PA, including walking, jogging, cycling, swimming, sports, and weight lifting, decreased. Additionally, parameters of SB, including time spent watching TV, using social media and electronics, increased among the majority of the participants. According to the results, age, gender, income, occupation, obesity, and being worried about conducting COVID-19 seem to predict the changes in PA and SB participation. Given the novelty of the disease and the sparsity of information, it is difficult to explain the current results. Previous studies have suggested cultural,^40^ psychological,^41^ behavioral,^42^ biological,^43^ environmental,^44^ economic,^45^ and policy^46^ detriments of participating in PA. The importance of these factors seems to vary across social segments, age, gender, location and education.^40^–^46^ Therefore, more studies are needed to verify the current findings under confinement situations, especially due to infectious diseases. Subsequently, design studies and strategies to improve exercise adherence among adults during disease-induced confinement.

### Implications

As previously has been anticipated,^29^–^34^ PA decreased, whereas SB increased among the participants in the current study during confinement due to COVID-19. The ramifications of these changes remained to be unraveled. However, the adverse health effects of decreased PA and increased SB are well documented.^29^–^34^ Therefore, strategies are needed to help people staying active and to mitigate the possible adverse health effect during the current and future pandemics.

### Limitations

The design of the current study is cross-sectional, which makes concluding a cause-effect relationships difficult. The measures of PAs and SBs are crude without details of the intensity, duration, frequency, progression, and METs. Additionally, the inherited misinformation in self-reported survey research is a disadvantage of the current study. Furthermore, the study participants are from Jordan, which confines the generalizability of the results in other communities, countries, and ethnicities. Therefore, future interventions and longitudinal studies in different regions of the world using objective measures of PAs and SBs are warranted to better understand the effect of confinement during the current and future pandemics.

## Conclusions

The current study revealed a 35.9–44.5% decrease in the different modes of PAs and a 72.1–82.7% increase in the various SB measures. Additionally, age, gender, income, occupation, obesity, and being worried about conducting COVID-19 are associated with the changes in PA and SB. However, studies are needed to verify the current findings and strategies are warranted to encourage people staying active and to mitigate the adverse health effects of inactivity due to confinement during the current and future pandemics.
